# Association of *PPARGC1A* Gly428Ser (rs8192678) polymorphism with potential for athletic ability and sports performance: A meta-analysis

**DOI:** 10.1371/journal.pone.0200967

**Published:** 2019-01-09

**Authors:** Phuntila Tharabenjasin, Noel Pabalan, Hamdi Jarjanazi

**Affiliations:** 1 Chulabhorn International College of Medicine, Thammasat University, Pathum Thani, Thailand; 2 Environmental Monitoring and Reporting Branch, Ontario Ministry of the Environment Conservation and Parks, Toronto, Ontario, Canada; GeneDx, UNITED STATES

## Abstract

**Background:**

Genetics plays a role in determining potential for athletic ability (AA) and sports performance (SP). In this study, AA involves comparing sedentary controls with competitive athletes in power and endurance activities as well as a mix between the two (SP). However, variable results from genetic association studies warrant a meta-analysis to obtain more precise estimates of the association between *PPARGC1A* Gly482Ser polymorphism and AA/SP.

**Methods:**

Multi-database literature search yielded 14 articles (16 studies) for inclusion. Pooled odds ratios (ORs) and 95% confidence intervals (CI) were used to estimate associations. Summary effects were modified based on statistical power. Subgroup analysis was based on SP (power, endurance and mixed) and race (Caucasians and Asians). Heterogeneity was assessed with the I^2^ metric and its sources examined with outlier analysis which dichotomized our findings into pre- (PRO) and post-outlier (PSO).

**Results:**

Gly allele effects significantly favoring AA/SP (OR > 1.0, P < 0.05) form the core of our findings in: (i) homogeneous overall effect at the post-modified, PSO level (OR 1.13, 95% CI 1.03–1.25, P = 0.01, I^2^ = 0%); (ii) initially homogeneous power SP (ORs 1.22–1.25, 95% CI 1.05–1.44, P = 0.003–0.008, I^2^ = 0%) which precluded outlier treatment; (iii) PRO Caucasian outcomes (ORs 1.29–1.32, 95% CI 1.12–1.54, P = 0.0005) over that of Asians with a pooled null effect (OR 0.99, 95% CI 0.72–1.99, P = 0.53–0.92) and (iv) homogeneous all > 80% (ORs 1.19–1.38, 95% CI 1.05–1.66, P = 0.0007–0.007, I^2^ = 0%) on account of high statistical power (both study-specific and combined). In contrast, none of the Ser allele effects significantly favored AA/SP and no Ser-Gly genotype outcome favored AA/SP. The core significant outcomes were robust and showed no evidence of publication bias.

**Conclusion:**

Meta-analytical applications in this study generated evidence that show association between the Gly allele and AA/SP. These were observed in the overall, Caucasians and statistically powered comparisons which exhibited consistent significance, stability, robustness, precision and lack of bias. Our central findings rest on association of the Gly allele with endurance and power, differentially favoring the latter over the former.

## Introduction

Athletic ability (AA) determines sports performance (SP). SP is a highly polygenic and complex phenotype as well as having a multifactorial etiology where genetic and environmental factors contribute to differences among trained athletes [1]. In this study, the role of genetics in determining AA is examined in the context of strength/power and endurance activities as well as a mix between the two (SP). The mix represents a continuum of SP activities between power and endurance. Explosive activities such as sprint and weightlifting characterize strength/power while endurance involves sustained activities such as marathons and cycling. Muscle strength and sprint activity characterize power phenotypes while that of endurance is maximal oxygen uptake and economy of movement [2]. Muscle fiber composition in these two types of athletes differs where activation of types II and I fibers occur during high-intensity activity in power and endurance performances, respectively [3, 4]. These contrasts stem from diverging genetic backgrounds of power and endurance athletes that drive their physiology into different trajectories [5].

Multiple genetic variants are thought to influence muscle function and SP phenotypes [2]. Among the genetic loci associated with SP, *peroxisome proliferator-activated receptor gamma co-activator-1-alpha* (*PPARGC1A* or PGC-1 α) aroused interest for the varied functions of the proteins it encodes. *PPARGC1A* is encoded by the gene *PPARGC1A* in humans which is crucial in training-induced muscle adaptation because it co-activates a span of transcriptional factors that control myriad biological responses [6]. Studies have shown that several amino acid polymorphic sites exist within the coding region of *PPARGC1A*, including Gly482Ser (rs8192678), which is reported to have functional relevance [7].

Current understanding of Gly482Ser in *PPARGC1A* is viewed in terms of its impact on health (e.g. diabetes and obesity) and on athletic phenotype (e.g. endurance sports). In terms of health impact, physiological evidence has shown that Gly482Ser affects blood lipid levels and insulin sensitivity. Compared with carriers of 482Gly, those with 482Ser have higher levels of low density lipoprotein cholesterol [8] and higher insulin resistance [9]. As a result, such persons have increased risk for Type 2 diabetes [10, 11]. A number of similar and variable findings on the role of Gly482Ser in disease have been reported in the literature that warranted coverage in published meta-analyses [12–14]. Thus, current knowledge shows that studies on this polymorphism focused more on health outcomes rather than SP.

For the studies that focus on the association of *PPARGC1A* with SP, Gly482Ser has been regarded as a promising genetic polymorphism in determining SP status for both power and endurance-type athletes [2]. This promise may likely also determine AA. Information on why Gly482Ser may predispose to AA is mainly derived from studies that focused on differential genotype frequencies between athletes and controls, with findings that tend more toward endurance over that of power. This polymorphism was posited to be the genetic factor that predetermines aerobic capacity [15]. However, discrepancies among the primary study outcomes for Gly428Ser in SP hedge on the type of sport they refer to. For instance, the Ser allele has been found to be less frequent among elite athletes in endurance [15] and power [16]. Other studies, however, report the Ser allele as useful in power activities [17]. Thus, while the Ser allele was reported to disfavor endurance activities, the Gly allele was found useful [18, 19]. Regardless of exercise type, the Gly allele is considered important in athletics [19]. The reported advantage of Gly allele carriers confers value to this polymorphism in any polygenic athletic profile [20]. We performed this meta-analysis because reported findings on the role of Gly428Ser in SP have differed and the number of articles was ripe for synthesizing the variable findings.

Methodological problems may explain the variabilities, which include limited statistical power, unrecognized confounding factors, misleading definition of phenotypes and stratification of populations [21]. Thus, Gly482Ser studies in SP have been heterogeneous given the various research approaches, variable sample sizes and different population profiles that characterize them. Such heterogeneity then renders the proposed associations to be inconsistently replicated. Clearly, utilizing research methods that synthesize diverging primary study results is needed which meta-analysis seems most suitable to resolve. Nevertheless, to the best of our knowledge, this is the first meta-analysis to examine the role of Gly482Ser in determining AA/SP.

In this study, we focus on the genetic role of this polymorphism in AA/SP by using an array of meta-analytical techniques such as a scale to evaluate quality of the primary literature, tests of association, outlier and modifier treatments, sensitivity analysis and tests for publication bias in order to assess the strength of evidence. This in-depth treatment precludes covering other SP related genes. Thus, we view the single-polymorphism approach most suitable for reasons of brevity and clarity of reporting.

## Materials and methods

### Selection of studies

Three databases (MEDLINE using PubMed, Science Direct and Google Scholar) were searched for association studies as of June 15, 2018. Terms used were “*peroxisome proliferator-activated receptor-gamma co-activator 1-alpha”*, “*PPARGC1A*”, “PGC1-α”, “rs8192678”, “sports performance” and “polymorphism” as medical subject heading and text, restricted to the English language. Additional eligible studies were identified from references cited in the retrieved articles. Inclusion criteria were: (i) case–control study design evaluating the association between *PPARGC1A* polymorphisms and SP; (ii) studies comply with the Hardy-Weinberg Equilibrium (HWE); (iii) sufficient genotype or allele frequency data to allow calculation of odds ratios (ORs) and 95% confidence intervals (CIs). Exclusion criteria include: (i) studies that do not involve SP (e.g. *PPARGC1A* polymorphism effects in pathophysiological conditions such as diabetes or cases were non-athletes); (ii) studies whose genotype or allele frequencies were otherwise unusable / absent or when available but combined with other polymorphisms, preventing proper data extraction; (iii) in case of duplicates, we chose the most recent article; (iv) reviews; (v) no controls; (vi) when controls were present, their frequencies deviated from the HWE and (vii) non-human subjects and non-English articles.

### Data extraction

Two investigators (PT and NP) independently extracted the data and arrived at consensus. Extracted information from each article comprised of the following: first author’s name, publication year, country of origin and SP type. S1 Table tabulates information on the quantitative data. Core information here is of two types: (i) because the literature on *PPARGC1A* and SP compared athletes with sedentary controls, we examine propensity for AA, rather than SP in itself. However, SP in this study is contextualized in terms of power, endurance or mixed. (ii) Genotype data was used to conduct the meta-analysis, which precludes extraneous information such as environmental data which was either unmentioned or unquantified in the primary literature. Of note, HWE was assessed using the application in https://ihg.gsf.de/cgi-bin/hw/hwa1.pl. Authors were contacted in order to obtain more information on incomplete data.

### Cases and controls

Cases in the included studies were athletes who have participated in competitions (the type of competition is bracketed) which the component studies stratified into (i) top-elite or world-class [World and/or European Championships or Olympic Games]; (ii) sub-elite [National level] and (iii) non-elite [regional level]. However, presentation of the genotype data for *PPARGC1A* did not differentiate between these stratifications. Controls were generally healthy, sedentary, without competitive sports experience.

### Quality assessment of the studies

We used the Clark-Baudouin scale to assess methodological quality of the included studies [21]. This scale has criteria (P-values, statistical power, corrections for multiplicity, comparative sample sizes between cases and controls, genotyping methods and HWE) that are found in the component articles. In this scale, low, moderate and high have scores of < 5, 5–7 and ≥ 8, respectively.

### Meta-analysis

Gly482Ser associations with SP (OR) were estimated for each study. Presence of zero genotype values warranted application of the Laplace correction which involves adding a pseudo-count of one to all values of the data set [22] prior to generating the forest plots. We used the allele-genotype approach to enable comparison with study-specific outcomes. We thus compared the following for Gly482Ser: (i) Gly allele with Ser-Gly/Ser-Ser genotype; (ii) Ser allele with Ser-Gly/Gly-Gly genotype and (iii) Gly/Ser genotype with homozygous Gly-Gly and Ser-Ser genotypes. Comparing effects on the same baseline, we used raw data from genotype frequencies to calculate pooled ORs. Pooled ORs with their accompanying 95% CIs were used to assess the strength of evidence. These are: (i) magnitudes of effects are higher or lower when the values are farther from or closer to the OR value of 1.0 (null effect), respectively; (ii) OR values are significant when P < 0.05 (two-sided); (iii) distance from P < 0.05, where farther from (e.g. P < 0.0001) and closer to (e.g. P < 0.04) this value indicates stronger and weaker association, respectively and (iv) CID (confidence interval difference) results when the lower CI is subtracted from the upper CI, which indicates precision of effects. High and low CID values indicate low and high precision, respectively.

Heterogeneity between studies was estimated with the χ ^2^-based Q test [23], explored with subgroup analysis [23] and quantified with the I^2^ statistic which measures variability between studies [24]. The fixed effects model [25] was used when P ≥ 0.10 or I^2^ < 50%, otherwise we opted for the random effects model [26], signifying presence of heterogeneity. Sources of heterogeneity were detected with the Galbraith plot [27] followed by re-analysis. Three features of this re-analysis are worth noting: (i) outlier treatment dichotomizes the comparisons into pre-outlier (PRO) and post-outlier (PSO) which are integrated in the design of the summary tables; (ii) outlier treatment is applied in PRO which assumes the random-effects status and (iii) PSO outcomes are fixed-effects, where larger studies are accorded more weight [28]. The Bonferroni correction, applied to independent comparisons only, was used to adjust for multiple testing. Sensitivity analysis, which involves omitting one study at a time and recalculating the pooled OR, was used to test for robustness of the summary effects. Publication bias was assessed on comparisons with ≥ 10 studies only [29]. Data were analyzed using Review Manager 5.3 (Cochrane Collaboration, Oxford, England), SIGMASTAT 2.03, SIGMAPLOT 11.0 (Systat Software, San Jose, CA) and WINPEPI [30].

## Results

### Search results

**Fig 1:**
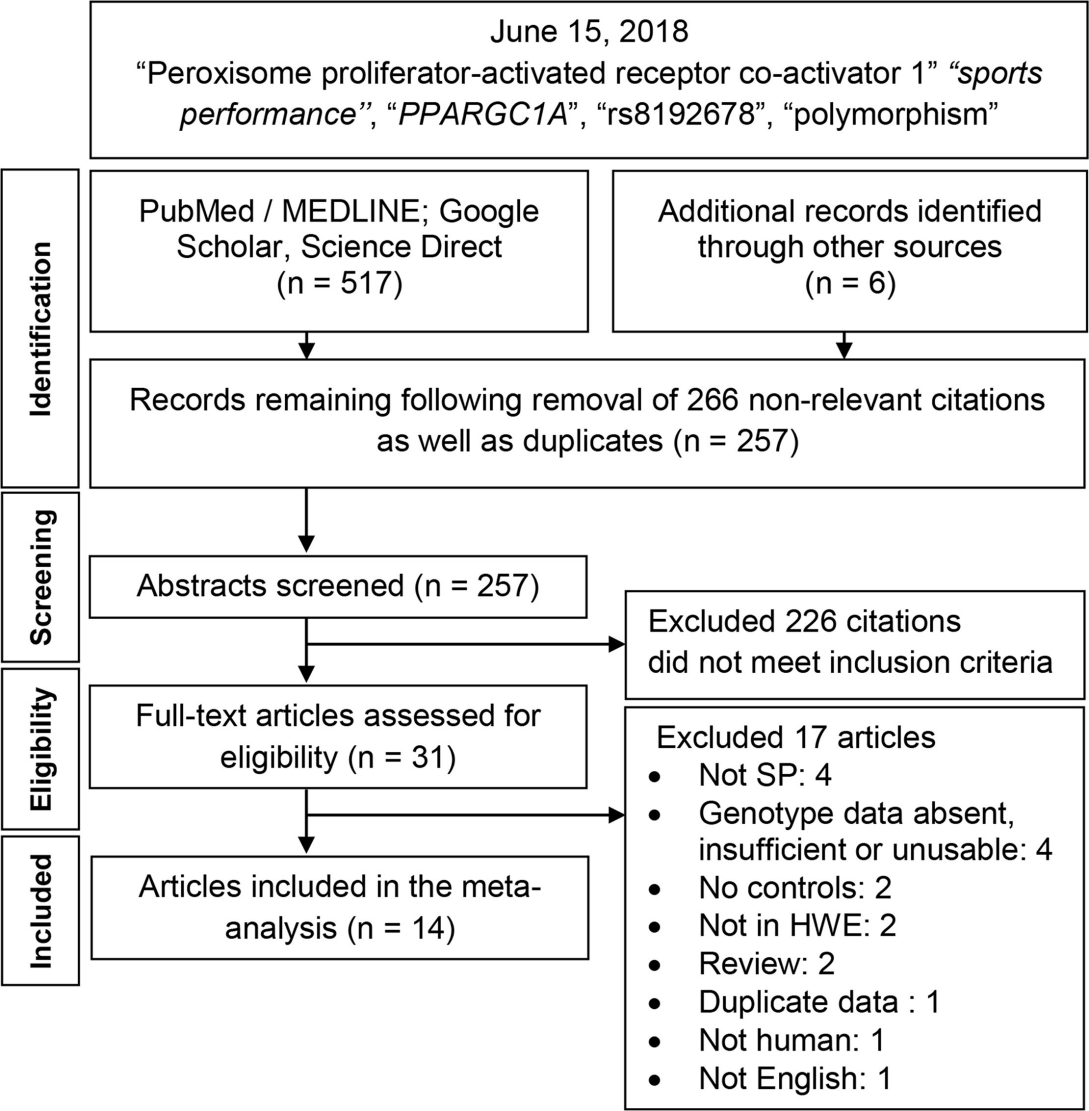
Summary flowchart of literature search. *PPARGC1A*: *Peroxisome proliferator-activated receptor gamma co-activator-1-alpha*; SP: sports performance; HWE: Hardy-Weinberg Equilibrium.

Fig 1 outlines the study selection process in a flowchart following PRISMA (Preferred Reporting Items for Systematic Reviews and Meta-Analyses) guidelines. Initial search resulted in 523 citations, followed by a series of omissions (S1 List) that eventually yielded 14 articles for inclusion [15–20, 31–38]. Of the 14, two articles [17, 19] presented independent data from two populations placing the total number of studies to 16 (Table 1).

**Table 1:** Characteristics of the included studies that examined the association of *PPARGC1A* Gly482Ser polymorphism with sports performance.

K	First author [Reference]		Year	n	Country	Race	SP status	Clark-Baudouin score
1	Ahmetov [31]		2009	1	Russia	C	Endurance	7
2	Eynon [32]		2011	1	Israel	C	Power/Endurance	8
3	Gineviciene [16]		2011	1	Lithuania	C	Power/Endurance/Mixed	9
4	Gineviciene [33]		2012	1	Lithuania	C	Mixed	7
5	Gineviciene [17]		2016	2	Lithuania/Russia	C	Power	8
6	Grealy [20]		2015	1	Australia	C	Endurance	7
7	He [34]		2014	1	China	A	Endurance	5
8	Jin [18]		2014	1	Korea	A	Mixed	7
9	Lucia [15]		2005	1	Spain	C	Endurance	7
10	Maciejewska [19]		2012	2	Poland/Russia	C	Power/Endurance/Mixed	7
11	Maruszak [35]		2012	1	Poland	C	Power/Endurance	5
12	Muniesa [36]		2010	1	Spain	C	Endurance	7
13	Peplonska [37]		2016	1	Poland	C	Mixed	7
14	Yvert [38]		2016	1	Japan	A	Endurance	8

K: number designation of the article; n: number of studies; C: Caucasian; A: Asian; SP: sports performance

### Characteristics of the included studies

Table 1 shows that participants in most of the studies were Euro-Slavic with three (Australia, Japan and Korea) contributing to geographical heterogeneity [18, 20, 38]. Subgroups by sport type and race comprised of power [16, 17, 19, 32, 35], endurance [15, 16, 19, 20, 31, 32, 34–36, 38] and mixed [16, 18, 19, 33, 37], Caucasian [15–17, 19, 20, 31–33, 35–37] and Asian [18, 34, 38], respectively. Median and range Clark-Baudouin score of 7.0 (5–9) indicates high methodological quality of the component studies. S2 Table shows that the meta-analysis is composed of seven, 11, and six studies in power, endurance, and mixed, respectively. Quantitative features include sample sizes, genotype frequencies in cases/controls and minor allele frequencies (maf) in each of the sport types (S2 Table). The maf means and standard deviations of Caucasians (0.34 ± 0.05) and Asians (0.49 ± 0.05) differed significantly (t = -4.86, P < 0.001). The checklists for PRISMA and meta-analysis for genetic association detailed features of this meta-analysis in accordance with the guidelines (S2 and S3 Tables).

### Meta-analysis outcomes

Table 2, S4 and S5 Tables summarize the meta-analysis outcomes by order of genetic comparisons (Gly and Ser alleles and Ser-Gly genotype). Between these three tables, number of pooled ORs > 1.0 (favoring SP) was most in Gly allele and least in Ser allele and none in Ser-Gly genotype. This positions the Gly allele analysis as central to our findings because it presents the most convincing evidence indicating the favoring of AA/SP.

**Table 2:** Outlier and modified effects for Gly allele associations with sports performance.

		Test of association				Test of heterogeneity				Test of association				Test of heterogeneity			Effect of outlier treatment (Fs)	
	n	OR	95% CI	P^a^	SP	P^b^	I^2^ (%)	AM	n	OR	95% CI	P^a^	SP	P^b^	I^2^ (%)	AM	Significance	Heterogeneity
		PRO								PSO								
All	16	**1.24**	**1.08–1.41**	0.002 *	Fs	0.0006	62	R	14	**1.16**	**1.06–1.26**	0.001 *	Fs	0.16	27	F	RS	RH
Power	7	**1.25**	**1.08–1.44**	0.003 *	Fs	0.66	0	F	—	——	——	——	—	—	—	—	—	—
Endurance	11	**1.24**	**1.02–1.51**	**0.03**	Fs	0.0005	68	R	8	**1.23**	**1.08–1.42**	**0.003**	Fs	0.10	41	F	ES	RH
Mixed	6	1.06	0.72–1.55	0.78	Fs	10^−4^	83	R	4	1.07	0.88–1.30	0.49	Fs	0.31	16	F	RNS	RH
***Race***																		
Caucasian	13	**1.29**	**1.12–1.49**	0.0005 *	Fs	0.002	61	R	11	**1.19**	**1.08–1.31**	0.0004 *	Fs	0.19	27	F	RS	RH
Asian	3	0.99	0.79–1.24	0.92	Null	0.34	8	F	—	——	——	——	—	—	—	—	—	—
***Modified***																		
All	10	**1.23**	**1.06–1.44**	**0.008**	Fs	0.001	67	R	8	**1.13**	**1.03–1.25**	**0.01**	Fs	0.53	0	F	RS	EH
All > 80%	5	**1.38**	**1.14–1.66**	0.0007 *	Fs	0.005	73	R	3	**1.19**	**1.05–1.34**	**0.007**	Fs	0.95	0	F	RS	EH
Power	6	**1.22**	**1.05–1.42**	**0.008**	Fs	0.64	0	F	—	——	——	——	—	—	—	—	—	—
Endurance	7	1.19	0.94–1.51	0.14	Fs	0.001	73	R	6	1.09	0.94–1.25	0.27	Fs	0.15	38	F	RNS	RH
Mixed	4	1.03	0.59–1.78	0.92	Fs	10^−5^	89	R	2	1.07	0.84–1.37	0.56	Fs	0.13	57	F	RNS	RH
***Race***																		
Caucasian	8	**1.32**	**1.13–1.54**	0.0005 *	Fs	0.009	63	R	6	**1.18**	**1.06–1.32**	**0.003**	Fs	0.78	0	F	RS	EH
Asian	2	0.99	0.72–1.99	0.53	Null	0.46	0	F	—	——	——	——	—	—	—	—	—	—

n: number of studies; Modified: ≥ 248 sample size in either case or control; All > 80%: studies with ≥ 248 participants in case and in control; PRO: pre-outlier; PSO: post outlier; OR: odds ratio; CI: confidence interval; P^a^: P-value for association

* P^a^ values that survived the Bonferroni correction; P^b^: P-value for heterogeneity; AM: analysis model; R: random-effects; F: fixed-effects; SP: sports performance; Fs: favor SP; ORs = 0.99–1.01 were considered null; RS: retained significance; RNS: retained non-significance; ES: elevated significance; RH: reduced heterogeneity; EH: eliminated heterogeneity. Values in bold indicate significant associations that favor SP only.

### Gly allele effects

Table 2 shows the Gly allele associations where 20 (91%) of the 22 comparisons favored AA/SP (OR >1.0). Of the 20 AA/SP favoring outcomes, 14 (70%) were statistically significant (P < 0.05). Of the 14 significant outcomes, half survived the Bonferroni correction, five in PRO and two in PSO. These AA/SP favoring and significant features were observed in the overall (ORs 1.16–1.24, 95% CI 1.06–1.41, P = 0.001–0.002) and Caucasian subgroup (ORs 1.19–1.29, 95% CI 1.08–1.49, P = 0.0004–0.0005). In contrast to Caucasians, the Asian effects were null and non-significant (OR 0.99, 95% CI 0.79–1.24, P = 0.92).

**Fig 2:**
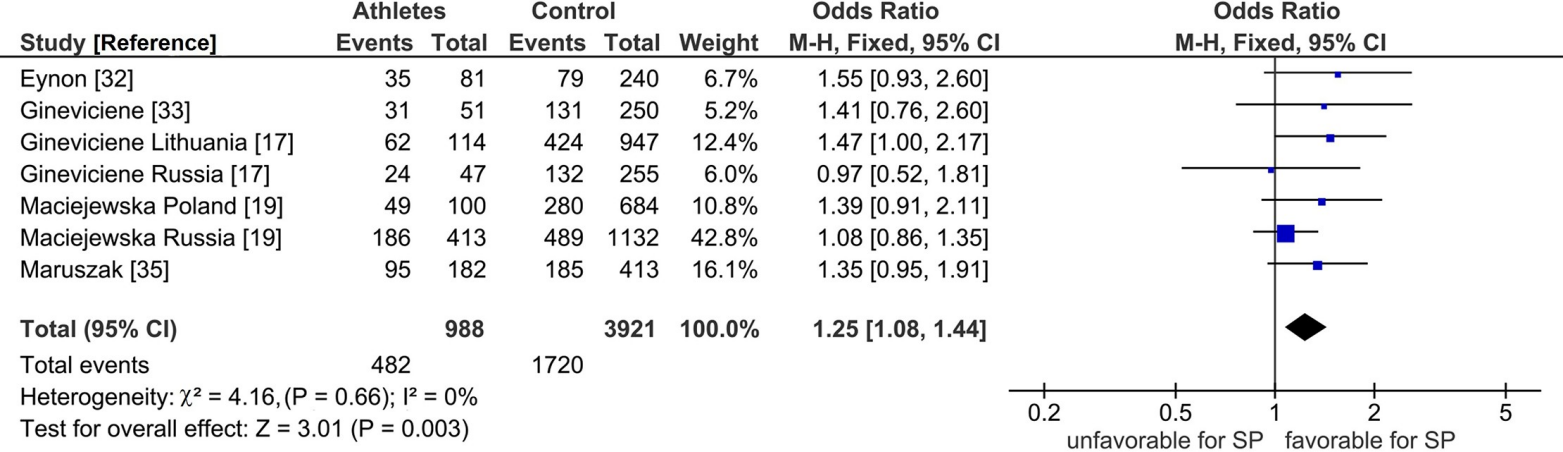
Forest plot outcome of *PPARGC1A* Gly allele effects on SP power in the pre-modifier analysis. Diamond denotes the pooled odds ratio (OR). Squares indicate the OR in each study, with square sizes directly proportional to the weight contribution (%) of each study. Horizontal lines represent 95% confidence intervals (CI). The Z test for overall effect indicates significance (P = 0.003). The χ^2^ test shows absence of heterogeneity (P = 0.66, I^2^ = 0%). LEGEND: M-H: Mantel-Haenszel; I^2^: measure of variability expressed in %.

Fig 2 and Table 2 delineate salient differences between power and endurance where both effects were significant (P < 0.05). The high significance in power (P = 0.003) survived the Bonferroni correction, but the moderate significance in endurance (P = 0.03) did not (Table 2).

In terms of pooled effects, that in power (OR 1.25, 95% CI 1.08–1.44) was initially homogeneous (I^2^ = 0%), while that in endurance (OR 1.24, 95% CI 1.02–1.51) was initially heterogeneous (I^2^ = 68%). Endurance heterogeneity warranted outlier treatment, but power homogeneity did not.

Outlier treatment had multiple effects on a number of parameters: (i) heterogeneity was reduced (P_heterogeneity_ ≥ 0.10) or eliminated (I^2^ = 0%); (ii) significance was retained (overall, Caucasian, all > 80%) and elevated (endurance) and (iii) precision of effects was increased (reduction of CID values from PRO to PSO).

**Fig 3:**
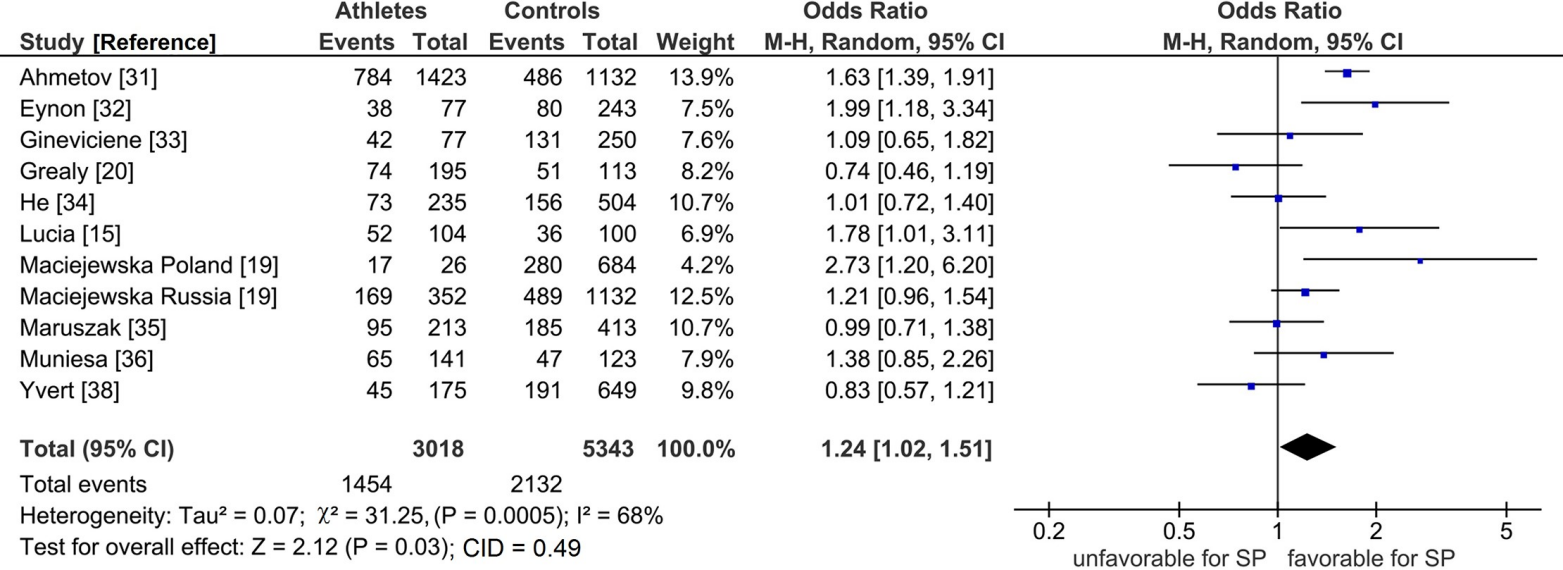
Forest plot outcome of *PPARGC1A* Gly allele effects on SP endurance in the PRO analysis. Diamond denotes the pooled odds ratio (OR). Squares indicate the OR in each study, with square sizes directly proportional to the weight contribution (%) of each study. Horizontal lines represent 95% confidence intervals (CI). The Z test for overall effect indicates significance (P = 0.03). The χ ^2^ test shows presence of heterogeneity (P = 0.0005, I^2^ = 68%). LEGEND: M-H: Mantel-Haenszel; I^2^: measure of variability expressed in %; CID, confidence interval difference.

**Fig 4:**
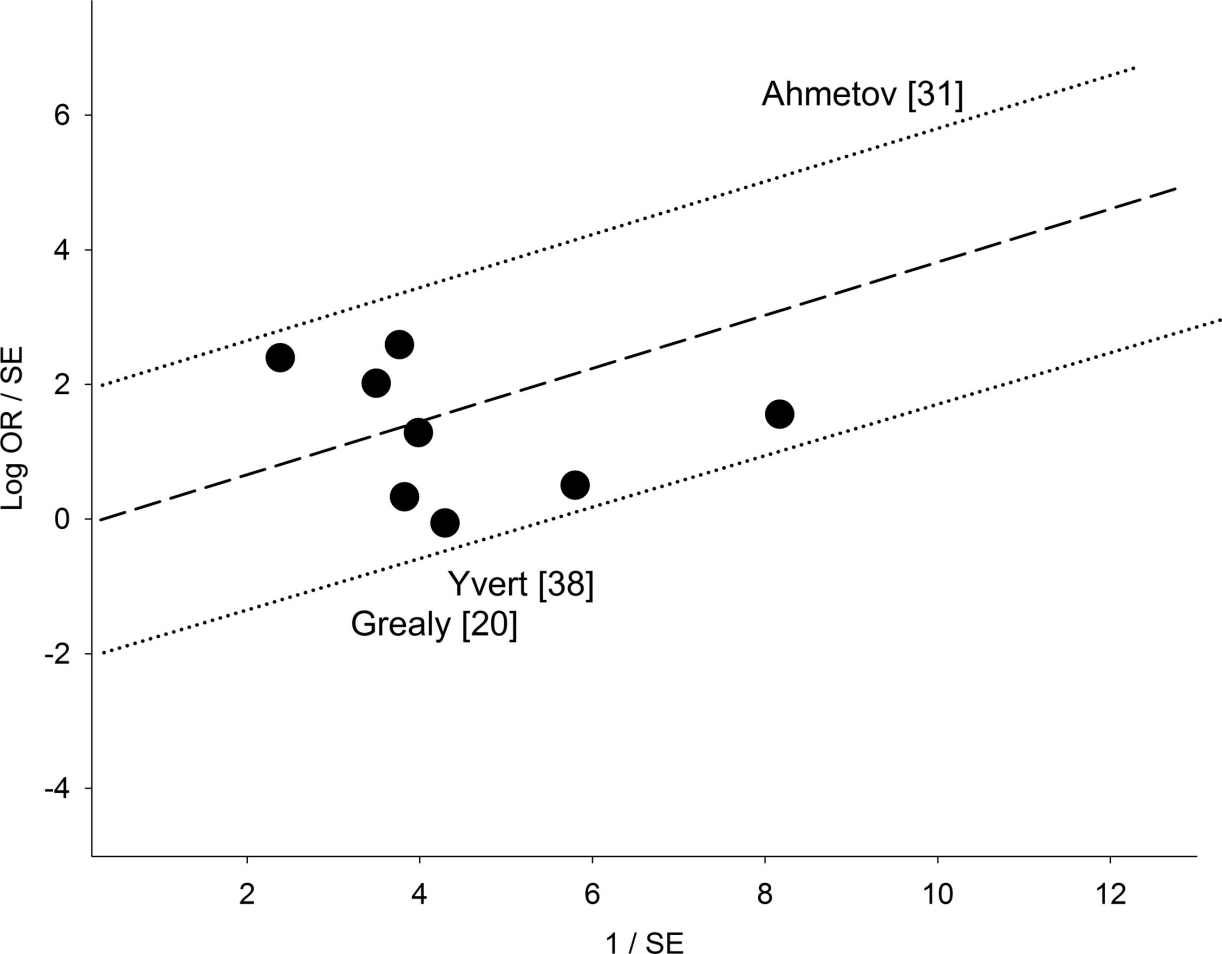
Galbraith plot analysis of Gly allele effects on SP in endurance identifying the sources of heterogeneity. The three studies that lie above and below the +2 and—2 confidence limits are the outliers. LEGEND: Log OR: logarithm of standardized odds ratio; SE: standard error.

**Fig 5:**
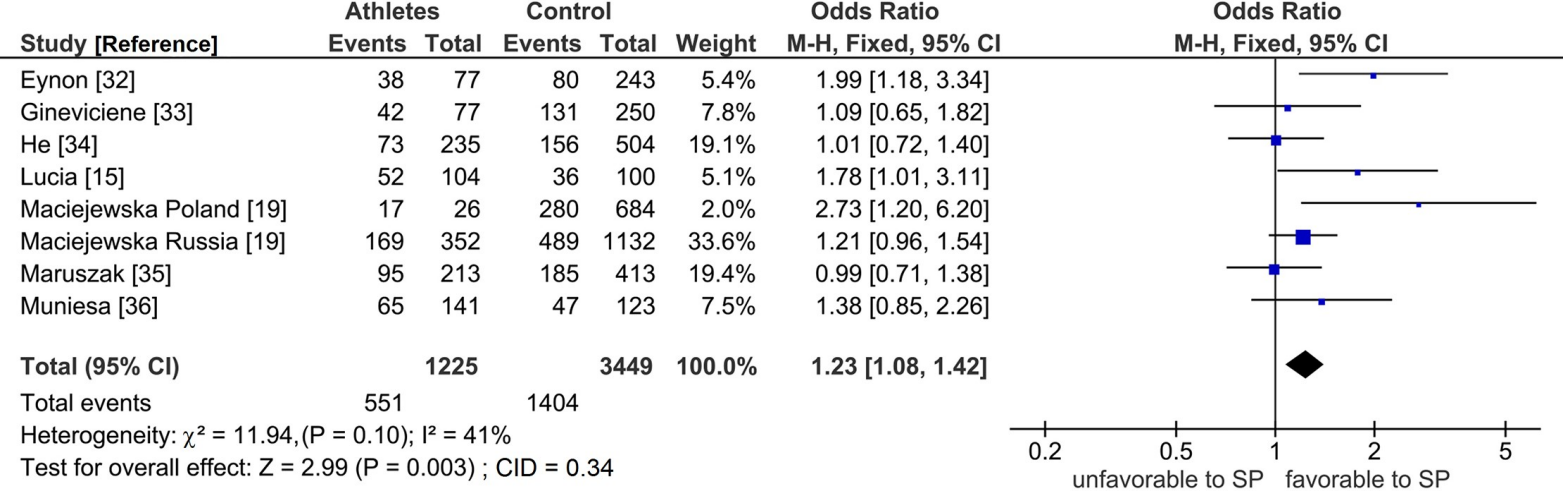
Forest plot outcome of outlier treatment on *PPARGC1A* Gly allele effects on SP endurance in the PSO analysis. Diamond denotes the pooled odds ratio (OR). Squares indicate the OR in each study, with square sizes directly proportional to the weight contribution (%) of each study. Horizontal lines represent 95% confidence intervals (CI). The Z test for overall effect indicates high significance given (P = 0.003). The χ ^2^ test indicates non-heterogeneity (P = 0.10, I^2^ = 41%). LEGEND: M-H: Mantel-Haenszel; I^2^: measure of variability expressed in %; CID, confidence interval difference.

The mechanism of outlier treatment is visualized in Figs 3–5. Fig 3 shows the following features for Gly allele endurance PRO: (i) heterogeneous (P_heterogeneity_ = 0.0005, I^2^ = 68%); (ii) moderately significant (OR 1.24, 95% CI 1.02–1.51, P = 0.03) and (iii) CID of 0.49 (CI 1.02–1.51). In Fig 4, the Galbraith plot identifies three studies as the outliers [20, 31, 38] located above the +2 and below the -2 confidence limits. In Fig 5, the PSO outcome (outliers omitted) shows (i) reduced heterogeneity (P_heterogeneity_ = 0.10, I^2^ = 41%); (ii) increased significance (OR 1.23, 95% CI 1.08–1.42, P = 0.003) and (iii) increased precision with a reduced CID of 0.34 (CI 1.08–1.42). This operation is numerically summarized in Table 2.

### Modified effects in Gly comparisons

Testing a single nucleotide polymorphism using a case-control design has been calculated to require a sample size of 248 participants in each group to achieve a statistical power of 80% [39]. To approximate this level and still achieve enough studies, we selected those with at least 248 participants in either cases or controls for Gly allele comparison only. However, we also included five studies from four papers [19, 31, 35, 37] with > 248 participants both in cases and in controls which we termed “all > 80%”. Using the G*Power program [40], statistical powers in each of these three studies were calculated to range between 81.0% and 99.9% assuming an α level of 5%. Outcomes of all > 80% not only significant (ORs 1.19–1.38, 95% CI 1.05–1.66, P = 0.0007–0.007) but homogeneous (I^2^ = 0%) at the PSO level.

In Table 2, modified analysis further highlighted the differences between power and endurance effects. Modified power outcomes (OR 1.22, 95% CI 1.05–1.42, P = 0.008) reflected the overall effect indicating consistency of significance for this SP type. In contrast, modified endurance effect lost significance (OR 1.19, 95% CI 0.94–1.51, P = 0.14) when compared to the overall outcome. Outlier application to the modified and heterogeneous (I^2^ = 73%) endurance pooled effect reduced heterogeneity (I^2^ = 38%) and retained non-significance (P = 0.27).Other comparisons (PRO and PSO) reduced and eliminated heterogeneity (Overall, Caucasian and Asia: I^2^ = 8–27% to 0%).

### Ser allele and Ser-Gly genotype effects

S4 Table shows the Ser allele associations where three (14%) of the 22 comparisons (PRO and PSO) favored SP (ORs 1.08–1.13, 95% CI 0.83–1.44). Of the three, none were significant (P > 0.05). Eighteen of the 22 (82%) comparisons in PRO and PSO disfavored SP (ORs 0.57–0.95, 95% CI 0.41–1.23) of which, two (11%) were significant (P < 0.05). The remaining comparison was the null Asian effect (OR 1.01, 95% CI 0.79–1.31, P = 0.91).

S5 Table shows the Ser-Gly genotype associations 13 outcomes of which 11 (85%) disfavored SP (ORs 0.80–0.87, 95% CI 0.69–1.00, P < 10^−4^–0.06) and two (15%) had null (Asian) outcomes (ORs 1.00–1.01, 95% CI 0.80–1.26, P = 0.95–1.00). All Ser-Gly genotype comparisons had the fixed-effects feature indicating initial non-heterogeneity.

### Sensitivity analysis and publication bias

Sensitivity analysis was performed using a modified protocol that confined this treatment to the significant Gly allele findings. Pooled effects that retained (P < 0.05) or lost (P > 0.05) significance were considered robust and not robust, respectively. Table 3 identifies the robust and non-robust comparisons. The most robust comparisons were overall and all > 80% in the PRO analysis and all Caucasian outcomes. The PRO analysis had seven robust outcomes and as many interfering studies; PSO had half the number of robust outcomes and one unduplicated interfering study [19]. All unstable comparisons are attributed to six studies from five articles [15, 19, 31, 32, 36]. Our sensitivity PRO findings highlight the difference between the SP types where power outcome was robust and endurance was not. Data (study-specific ORs) used to test for publication bias was determined to be normally distributed (Kolmogorov-Smirnov test: P > 0.05). Hence, we used the Egger’s regression asymmetry test only which showed no evidence of publication bias (Table 4).

**Table 3:** Sensitivity analysis of Gly allele comparisons with significant outcomes favoring sports performance.

Comparison	PRO	PSO	Number of References contributing to non-robustness	Number of robust outcomes
Overall	Robust	Robust	0	2
Power	Robust	—-	0	1
Endurance	[15, 19, 31, 32, 36]	Robust	5	1
Caucasian	Robust	Robust	0	2
***Modified***				
Overall	Robust	[19]	1	1
All > 80%	Robust	[19]	1	1
Power	Robust	—-	0	1
Caucasian	Robust	Robust	0	2
Number of References contributing to non-robustness	5	2		
Number of robust outcomes	7	4		

PRO: pre-outlier; PSO: post-outlier; Numbers in brackets indicate references that contributed to non-robustness

**Table 4:** Publication bias assessment of Gly allele comparisons with significant outcomes favoring sports performance.

		Egger's regression asymmetry test	
	n	Intercept	P-value
All PRO	16	-1.15	0.25
All PSO	13	0.23	0.78
Endurance PRO	11	-0.84	0.52
Caucasian PRO	13	-0.93	0.39
Caucasian PSO	11	0.00	1.00

PRO: pre-outlier; PSO: post-outlier; n: number of studies; non-significant P-values (> 0.05) indicate absence of evidence of publication bias

## Discussion

### Summary of effects

In this meta-analysis, we present evidence of; (i) Gly allele outcomes favoring the potential for AA/SP over that of the Ser allele; (ii) within the Gly allele, Caucasians are affected but not Asians and (iii) Gly allele favors propensity for AA in power SP more than endurance and not in mixed sports at all. Of note, strength of the potential for power athletics lies in its homogeneity and stability of surviving the Bonferroni correction. (iv) Strength of the Gly allele effect is shown by the all > 80% outcomes showcasing the statistical power of this modified comparison.

Our findings delineated which genetic component of Gly428Ser in the *PPARGC1A* gene favored SP (Gly allele) and those that did not (Ser allele and Ser-Gly genotype). Subjecting these components to meta-analysis treatments (outlier, modified and sensitivity) impacted on the outputs. For example, the combined application of outlier and modifier treatments unraveled favorable features of the pooled outcomes that included reduced/ eliminated heterogeneity and elevated statistical power. Outlier treatment attempts to resolve heterogeneity issues that are inherent in meta-analysis. Modifier treatment operates through exclusion of underpowered studies. Underpowered outcomes appear to be common in candidate gene studies [28] and are prone to the risk of Type 1 error. This risk was addressed by generating a comparison with increased statistical power and correcting for multiple comparisons. Thus, all > 80% modified analysis was created especially in light of significant results [41] and Bonferroni correction to minimize the possibility of false-positive outcomes [42]. Both outlier and modifier treatments raise the levels of evidence presented here and highlight the transparency of our findings.

The main findings of this study center on the Gly allele on account of the following: (i) differential effects between power and endurance SP were clarified based on statistical (significance and correction) and meta-analytical treatments (modifier and sensitivity) and (ii) statistical significance were observed in all > 80% and the Caucasian subgroup. The SP favoring Gly allele bearing Caucasians but not Asians may be attributed to the significant difference in maf between the two races. While the Asian subgroup acquired zero heterogeneity on account of modifier treatment, the Caucasian subgroup acquired homogeneity on account of modifier and outlier treatments combined.

While homogeneous outcomes in meta-analysis improve the quality of evidence, heterogeneous results are unavoidable and must be addressed. A pro-active approach to addressing heterogeneity is identifying its sources using outlier treatment to re-analyze the results. Our application of outlier treatment had far-reaching effects, impacting on significance, heterogeneity and precision. However, it did not eliminate heterogeneity for the most part. Reduced heterogeneity, notwithstanding, our meta-analysis findings, such as those in the endurance outcomes agree with the physiological evidence [18, 19]. However, our meta-analysis results favor power more than endurance suggesting that *PPARGC1A* polymorphism may affect other physiological parameters related to power performance.

Variable pooled outcomes (ORs that skirt the null effect [ORs 0.99–1.01] and none that indicate favoring SP) observed in mixed sports effectively differs from the SP favoring power outcomes which seem to reflect the inherent phenotypic heterogeneity of this sport type [43]. Ser allele and Ser-Gly genotype effects were consistent in disfavoring SP, regardless of sport type and outlier treatment [19]. Favoring SP outcomes are underpinned by a number of important features: (i) similar repeated effects in the comparisons (consistency); (ii) reduced PSO heterogeneity (outcomes of outlier treatment); (iii) enhanced PSO significance (endurance); (iv) increased precision (reduced CID values from PRO to PSO) and (v) robustness (resistance to sensitivity treatment) all of which present strong evidence of Gly428Ser associations with SP.

### Genetic and physiological correlates

*PPARGC1A* has multiple physiological roles which include: (i) regulating cellular energy metabolism; (ii) regulating expression of genes that encode key enzymes involved in fatty acid oxidation [44] and oxidative phosphorylation [15]; (iii) it promoting glucose metabolism through upregulation of hepatic gluconeogenic genes [45, 46] and (iv) mediating skeletal muscle fiber type switching.

Combined peak force/power and ability to sustain high-intensity efforts for extended periods during a competition [4] is the process that uses oxidative metabolism [15, 19]. Skeletal muscle fiber type switching involves transition from glycolytic type IIb to mitochondria-rich types IIa and I which characterizes SP among power athletes. Mitochondrial amount in the recruited muscle fibers likely determines maximal sustainable power [47]. Not only has PPARGC1A been identified as master regulator of mitochondrial biogenesis, but it has also been shown to regulate proteins involved in angiogenesis and anti-oxidant defense as well as affect expression of inflammatory markers [19, 48]. The PPARGC1A protein has been shown to control muscle plasticity and suppress inflammatory response [7]. Acute exercise induces oxidative stress, mobilizes inflammatory response bolstering higher expression of *PPARGC1A* that may facilitate endurance athletes’ SP [49–51]. Expression levels of *PPARGC1A* have been shown to be altered in response to physiological stress or increased energy demands elicited by exercise training [52]. Thus, increase in *PPARGC1A* mRNA levels and its over-expression corresponds with delayed fatigue of the contracting muscle [53, 54] and this increase during exercise [54, 55] enhances skeletal muscle oxidative capacity [32, 53, 56]. Investigators have examined the role of *PPARGC1A* mRNA expression in SP where its levels were impacted by exercise training in both mouse and human skeletal muscle [43]. In mouse muscle, *PPARGC1A* is required to uphold mitochondrial protein expression which is needed for oxidative phosphorylation and perturbation of this cascade results in diminished exercise capacity [57]. In humans, Mathai et al. demonstrated that one session of protracted endurance activity induces elevated transcription and mRNA levels of *PPARGC1A* [46]. *PPARGC1A* has been shown to be expressed at high levels in metabolically active tissues where mitochondria are abundant and oxidative phosphorylation is operational such as brown adipose tissue, heart, and skeletal muscle, whereas expression level is low in white adipose tissue, liver, and pancreas [45, 58]. Functionality of the Gly482Ser polymorphism could likely affect mRNA expression and/or protein levels [32]. Therefore, knowledge of genotype may predict AA/SP [15]. Thus, *PPARGC1A* is implicated in promoting gene expression and muscle morphology characteristic of type I oxidative fibers in skeletal muscle.

### Strengths and limitations

Interpreting our findings here is best done in the context of its limitations and strengths. Limitations of our study include: (i) dominating presence of Slavic Caucasian participants (Russia, Lithuania). This precludes extrapolation of the findings to other ethnic groups. More studies are warranted to better represent a wider range of ethnic subgroups, particularly Asian populations; (ii) we did not examine female effects because of data unavailability. Only one [32] of the component studies presented gender-discriminating data which was insufficient to perform subgroup analysis. Although gender differences are not always clear, genes seem to play a more prominent role in male than in female strength determination [59]; (iii) most of the component studies were underpowered; (iv) heterogeneity of the PRO findings; (v) elevated statistical power through modified treatment was countered by non-robustness in the PSO analysis of overall and all > 80% and (vi) caution maybe warranted in concluding strong associations of the Gly allele in our study, given the possibility that this SP increasing allele may be in linkage disequilibrium with the true functional allele [60].

On the other hand, the following strengths not only add to the epidemiological, clinical and statistical homogeneity (hence, combinability) of the studies, but also minimize bias and underpin the magnitude of associations: (i) the combined sample sizes of the overall and SP types yielded high statistical power (S1 Table); (ii) screening for studies whose controls deviated from HWE effectively corrected for genotyping errors, which minimizes methodological weaknesses [61]; (iii) overall methodological quality (determined by the Clark-Baudouin score) of the included studies was high; (iv) outlier treatment reduce and eliminated heterogeneity. Impact of this treatment on significance and precision is viewed to favor our findings; (v) the PRO overall and power outcomes withstood the Bonferroni correction minimizing the possibility of a Type 1 error; (vi) sensitivity treatment conferred robustness to all overall and Caucasian findings, as well as modified overall and all > 80% in PRO and (vii) absence of evidence of publication bias nullifies the notion that it inflates significant pooled outcomes against non-significant results [28].

### Conclusions

We should point out that interpreting effects of polymorphisms differ between disease and SP, besides their respective domains in pathology and in normal phenotype. Disease effects are viewed in terms of protection (reduced risk) or susceptibility (increased risk) both of which have equal importance especially when significant. Potential for AA/SP effects on the other hand, is better contextualized when interpreting outcomes that favor SP (OR > 1.0). Thus, our reason for de-emphasizing ORs < 1.0 is that these values disfavoring AA/SP do not contribute to promoting AA/SP.

Highlights of our findings rests on the fact that most studies in this study lacked statistical power (68.8% in the overall analysis), but when the data are combined using meta-analysis, clear Gly allele effects are uncovered. We recognize that complexity of athletic potential involves interactions between genetic and non-genetic factors allowing for the possibility of environmental involvement in modifying Gly482Ser effects. Gene-gene and gene-environment interactions have been reported to have roles in associations of *PPARGC1A* polymorphisms with SP [5, 17]. While all but one [32] of the 11 articles mentioned gene-environment interaction, only two addressed haplotype analysis [19, 31]. Nevertheless, all but two [15, 19] analyzed polymorphisms in other genes, the most common being angiotensin converting enzyme and α-actinin in six [16, 17, 20, 31, 33, 36] and four articles [17, 20, 33, 36], respectively.

Including other SP-related genes in our meta-analysis would have been logistically problematic. Additional well-designed studies (including meta-analyses) exploring other parameters would confirm or modify our results in this study and add to the extant knowledge about the association of *PPARGC1A* polymorphism and potential for SP.

## Supporting information

S1 List Excluded articles. (DOCX)

S1 Table Quantitative features. (DOCX)

S2 Table PRISMA checklist. (DOCX)

S3 Table Checklist meta-analysis on genetic associations. (DOCX)

S4 Table Ser allele summary outcomes. (DOCX)

S5 Table Gly-Ser genotype summary outcomes. (DOCX)

## Abbreviations

A: Asian
AM: Analysis model
C: Caucasian
CI: Confidence interval
CID: Confidence interval difference
Ds: Disfavoring SP
EH: Eliminated heterogeneity
ES: Elevated significance
F: Fixed-effects
Fs: Favoring SP
Gly: Glycine
GS: Gain in significance
HWE: Hardy-Weinberg Equilibrium
I^2^: Measure of variability expressed in %
K: number designation of the article
Log OR: Logarithm of standardized OR
Maf: Minor allele frequency
M-H: Mantel-Haenszel
n: Number of studies
OR: Odds ratio
P: P-value
P^a^: P-value for association
P^b^: P-value for heterogeneity
PPARGC1A: Peroxisome proliferator-activated receptor gamma co-activator-1-alpha
PRISMA: Preferred Reporting Items for Systematic Reviews and Meta-Analyses
PRO: Pre-outlier
PSO: Post-outlier
R: Random-effects
RH: Reduced heterogeneity
RNS: Retained non-significance
RS: Retained significance
SE: Standard error
Ser: Serine
SP: Sports performance
