# Correction: Association of *PPARGC1A* Gly482Ser (rs8192678) polymorphism with potential for athletic ability and sports performance: A meta-analysis

**DOI:** 10.1371/journal.pone.0251672

**Published:** 2021-05-06

**Authors:** Phuntila Tharabenjasin, Noel Pabalan, Hamdi Jarjanazi

The genetic polymorphism “Gly482Ser” is misspelled in the article title, as well as in the Introduction, Discussion and Supporting Information. In all these cases, “Gly428Ser” should be “Gly482Ser”. The correct title is: Association of *PPARGC1A* Gly482Ser (rs8192678) polymorphism with potential for athletic ability and sports performance: A meta-analysis. The correct citation is: Tharabenjasin P, Pabalan N, Jarjanazi H (2019) Association of *PPARGC1A* Gly482Ser (rs8192678) polymorphism with potential for athletic ability and sports performance: A meta-analysis. PLoS ONE 14(1): e0200967. https://doi.org/10.1371/journal.pone.0200967
