# Supplementary material for: Association of PPARGC1A Gly428Ser (rs8192678) polymorphism with potential for athletic ability and sports performance: A meta-analysis
Source: PLoS One. 2019 Jan 9;14(1):e0200967. doi: 10.1371/journal.pone.0200967 (PMC6326506; doi:10.1371/journal.pone.0200967)
Supplement: S1 List — (DOCX) [file pone.0200967.s001.docx]

**S1 List. Excluded articles (n = 17)**

***Not related to competitive sports performance (n = 4)***

1. Garatachea N SC, Yvert T, Verde-Rello Z, Fiuza-Luces C, Santos-Lozano A, Gómez-Gallego F, Lucía A. Genetic variants in the PPARD-PPARGC1A-NRF-TFAM mitochondriogenesis pathway are neither associated with muscle characteristics nor physical performance in elderly. Revista Internacional de Ciencias del Deporte. 2015;11(41):196-208. doi: 10.5232/ricyde2015.04101.
2. Nishida Y, Iyadomi M, Higaki Y, Tanaka H, Kondo Y, Otsubo H, et al. Association between the PPARGC1A polymorphism and aerobic capacity in Japanese middle-aged men. Internal medicine. 2015;54(4):359-66. doi: 10.2169/internalmedicine.54.3170.
3. Sarzynski MA, Rankinen T, Sternfeld B, Grove ML, Fornage M, Jacobs DR, Jr., et al. Association of single-nucleotide polymorphisms from 17 candidate genes with baseline symptom-limited exercise test duration and decrease in duration over 20 years: the Coronary Artery Risk Development in Young Adults (CARDIA) fitness study. Circulation Cardiovascular genetics. 2010;3(6):531-8. doi: 10.1161/CIRCGENETICS.110.957183.
4. Steinbacher P, Feichtinger RG, Kedenko L, Kedenko I, Reinhardt S, Schonauer AL, et al. The single nucleotide polymorphism Gly482Ser in the PGC-1alpha gene impairs exercise-induced slow-twitch muscle fibre transformation in humans. PloS one. 2015;10(4):e0123881. doi: 10.1371/journal.pone.0123881.

***Genotype data absent or insufficient or derived (n = 4)***

1. Eynon N, Birk R, Meckel Y, Lucia A, Nemet D, Eliakim A. Physiological variables and mitochondrial-related genotypes of an athlete who excels in both short and long-distance running. Mitochondrion. 2011;11(5):774-7. doi: 10.1016/j.mito.2011.05.009.
2. Eynon N, Meckel Y, Alves AJ, Yamin C, Sagiv M, Goldhammer E, et al. Is there an interaction between PPARD T294C and PPARGC1A Gly482Ser polymorphisms and human endurance performance? Experimental physiology. 2009;94(11):1147-52. doi: 10.1113/expphysiol.2009.049668.
3. Ruiz JR, Gomez-Gallego F, Santiago C, Gonzalez-Freire M, Verde Z, Foster C, et al. Is there an optimum endurance polygenic profile? The Journal of physiology. 2009;587(Pt 7):1527-34. doi: 10.1113/jphysiol.2008.166645.
4. Santiago C, Ruiz JR, Muniesa CA, Gonzalez-Freire M, Gomez-Gallego F, Lucia A. Does the polygenic profile determine the potential for becoming a world-class athlete? Insights from the sport of rowing. Scandinavian journal of medicine & science in sports. 2010;20(1):e188-94. doi: 10.1111/j.1600-0838.2009.00943.x.

***No controls (n = 2)***

1. He Z, Hu Y, Feng L, Bao D, Wang L, Li Y, et al. Is there an association between PPARGC1A genotypes and endurance capacity in Chinese men? Scandinavian journal of medicine & science in sports. 2008;18(2):195-204. doi: 10.1111/j.1600-0838.2007.00648.x.
2. Tsianos GI, Evangelou E, Boot A, Zillikens MC, van Meurs JB, Uitterlinden AG, et al. Associations of polymorphisms of eight muscle- or metabolism-related genes with performance in Mount Olympus marathon runners. Journal of applied physiology. 2010;108(3):567-74. doi: 10.1152/japplphysiol.00780.2009.

***Not in Hardy-Weinberg Equilibrium (n = 2)***

1. Egorova ES, Borisova AV, Mustafina LJ, Arkhipova AA, Gabbasov RT, Druzhevskaya AM, et al. The polygenic profile of Russian football players. Journal of sports sciences. 2014;32 (13): 1286-93. doi: 10.1080/02640414.2014.898853.
2. Tural E, Kara N, Agaoglu SA, Elbistan M, Tasmektepligil MY, Imamoglu O. PPAR-alpha and PPARGC1A gene variants have strong effects on aerobic performance of Turkish elite endurance athletes. Molecular biology reports. 2014;41(9):5799-804. doi: 10.1007/s11033-014-3453-6.

***Review (n = 2)***

1. Ahmetov, II, Fedotovskaya ON. Current Progress in Sports Genomics. Advances in clinical chemistry. 2015;70:247-314. doi: 10.1016/bs.acc.2015.03.003.
2. Gonzalez-Freire M, Santiago C, Verde Z, Lao JI, Oiivan J, Gomez-Gallego F, et al. Unique among unique. Is it genetically determined? British journal of sports medicine. 2009;43(4):307-9. doi: 10.1136/bjsm.2008.049809.

***Duplicate data (n =1)***

1. Eynon N, Meckel Y, Sagiv M, Yamin C, Amir R, Sagiv M, et al. Do PPARGC1A and PPARalpha polymorphisms influence sprint or endurance phenotypes? Scandinavian journal of medicine & science in sports. 2010;20(1):e145-50. doi: 10.1111/j.1600-0838.2009.00930.x.

***Not human (n = 1)***

1. Stephenson EJ, Stepto NK, Koch LG, Britton SL, Hawley JA. Divergent skeletal muscle respiratory capacities in rats artificially selected for high and low running ability: a role for Nor1? Journal of applied physiology. 2012;113(9):1403-12. doi: 10.1152/japplphysiol.00788.2012.

***Not English (n = 1)***

1. Akhmetov, II, Popov DV, Mozhaiskaia IA, Missina SS, Astratenkova IV, Vinogradova OL, et al. [Association of regulatory genes polymorphisms with aerobic and anaerobic performance of athletes]. Rossiiskii fiziologicheskii zhurnal imeni IM Sechenova. 2007;93(8):837-43.
