# Supplementary material for: Association of PPARGC1A Gly428Ser (rs8192678) polymorphism with potential for athletic ability and sports performance: A meta-analysis
Source: PLoS One. 2019 Jan 9;14(1):e0200967. doi: 10.1371/journal.pone.0200967 (PMC6326506; doi:10.1371/journal.pone.0200967)
Supplement: S1 Table — (DOCX) [file pone.0200967.s002.docx]

**S1 Table Quantitative characteristics of *PPARGC1A* studies examining associations with sports performance**

|  |  |  |  |  | Genotype frequencies | | | | | |  |  |
| --- | --- | --- | --- | --- | --- | --- | --- | --- | --- | --- | --- | --- |
|  |  | Sample sizes | | | Case | | | Control | | | |  |
| K | First author country | Case | Control | Total | Gly-Gly | Ser-Gly | Ser-Ser | Gly-Gly | Ser-Gly | Ser-Ser | maf in controls | HWE P-values |
| Overall (5,505 cases / 7,311 controls) | | | | | | | | | | | | |
| 1 | Ahmetov | 1423 | 1132 | 2555 | 784 | 544 | 95 | 486 | 512 | 134 | 0.35 | 0.96 |
| 2 | Eynon | 155 | 240 | 395 | 72 | 73 | 10 | 79 | 117 | 44 | 0.43 | 0.95 |
| 3 | Gineviciene 1 | 193 | 250 | 443 | 101 | 77 | 15 | 131 | 100 | 19 | 0.28 | 0.99 |
| 4 | Gineviciene 2 | 199 | 167 | 366 | 95 | 85 | 19 | 83 | 74 | 10 | 0.28 | 0.22 |
| 5 | Gineviciene 6Lithuania | 47 | 255 | 302 | 24 | 22 | 1 | 132 | 106 | 17 | 0.27 | 0.48 |
| 6 | Gineviciene 6Russia | 114 | 947 | 1061 | 62 | 35 | 17 | 424 | 416 | 107 | 0.33 | 0.75 |
| 7 | Grealy | 195 | 113 | 308 | 74 | 84 | 37 | 51 | 45 | 17 | 0.36 | 0.19 |
| 8 | He | 235 | 504 | 739 | 73 | 115 | 47 | 156 | 244 | 104 | 0.55 | 0.63 |
| 9 | Jin | 111 | 145 | 256 | 37 | 58 | 16 | 39 | 77 | 29 | 0.47 | 0.42 |
| 10 | Lucia | 104 | 100 | 204 | 52 | 43 | 9 | 36 | 48 | 16 | 0.41 | 1.00 |
| 11 | Maciejewska Poland | 302 | 684 | 986 | 170 | 115 | 17 | 280 | 314 | 90 | 0.36 | 0.89 |
| 12 | Maciejewska Russia | 1303 | 1132 | 2435 | 623 | 569 | 111 | 489 | 505 | 138 | 0.35 | 0.67 |
| 13 | Maruszak | 395 | 413 | 808 | 190 | 170 | 35 | 185 | 195 | 33 | 0.31 | 0.06 |
| 14 | Muniesa | 141 | 123 | 264 | 65 | 52 | 24 | 47 | 63 | 13 | 0.36 | 0.23 |
| 15 | Peplonska | 413 | 457 | 870 | 199 | 178 | 36 | 199 | 213 | 39 | 0.32 | 0.09 |
| 16 | Yvert | 175 | 649 | 824 | 45 | 87 | 43 | 191 | 324 | 134 | 0.46 | 0.87 |
|  |  |  |  |  |  |  |  |  |  |  |  |  |
| Power (988 cases / 3,921 controls) | | | | | | | | | | | | |
| 1 | Eynon | 81 | 240 | 321 | 35 | 36 | 10 | 79 | 117 | 44 | 0.43 | 0.95 |
| 2 | Gineviciene 1Lithuania | 51 | 250 | 301 | 31 | 18 | 2 | 131 | 100 | 19 | 0.28 | 0.99 |
| 3 | Gineviciene 6Lithuania | 47 | 255 | 302 | 24 | 22 | 1 | 132 | 106 | 17 | 0.27 | 0.48 |
| 4 | Gineviciene 6Russia | 114 | 947 | 1061 | 62 | 35 | 17 | 424 | 416 | 107 | 0.33 | 0.75 |
| 5 | Maciejewska Poland | 100 | 684 | 784 | 49 | 42 | 9 | 280 | 314 | 90 | 0.36 | 0.89 |
| 6 | Maciejewska Russia | 413 | 1132 | 1545 | 186 | 188 | 39 | 489 | 505 | 138 | 0.35 | 0.67 |
| 7 | Maruszak | 182 | 413 | 595 | 95 | 70 | 17 | 185 | 195 | 33 | 0.31 | 0.06 |
|  |  |  |  |  |  |  |  |  |  |  |  |  |
| Endurance (3,018 cases / 5,343 controls) | | | | | | | | | | | | |
| 1 | Ahmetov | 1423 | 1132 | 2555 | 784 | 544 | 95 | 486 | 512 | 134 | 0.35 | 0.96 |
| 2 | Eynon * | 77 | 243 | 320 | 38 | 38 | 1 | 79 | 117 | 44 | 0.43 | 0.90 |
| 3 | Gineviciene 1Lithuania | 77 | 250 | 327 | 42 | 30 | 5 | 131 | 100 | 19 | 0.28 | 0.99 |
| 4 | Grealy | 195 | 113 | 308 | 74 | 84 | 37 | 51 | 45 | 38 | 0.36 | 0.19 |
| 5 | He | 235 | 504 | 739 | 73 | 115 | 47 | 156 | 244 | 104 | 0.55 | 0.63 |
| 6 | Lucia | 104 | 100 | 204 | 52 | 43 | 9 | 36 | 48 | 16 | 0.41 | 1.00 |
| 7 | Maciejewska Poland | 26 | 684 | 710 | 17 | 8 | 1 | 280 | 314 | 90 | 0.36 | 0.89 |
| 8 | Maciejewska Russia | 352 | 1132 | 1484 | 169 | 155 | 28 | 489 | 505 | 138 | 0.35 | 0.67 |
| 9 | Maruszak | 213 | 413 | 626 | 95 | 100 | 18 | 185 | 195 | 33 | 0.31 | 0.06 |
| 10 | Muniesa | 141 | 123 | 264 | 65 | 52 | 24 | 47 | 63 | 13 | 0.36 | 0.23 |
| 11 | Yvert | 175 | 649 | 824 | 45 | 87 | 43 | 191 | 324 | 134 | 0.46 | 0.87 |
|  |  |  |  |  |  |  |  |  |  |  |  |  |
| Mixed (1,502 cases / 2,835 controls) | | | | | | | | | | | | |
| 1 | Gineviciene 1Lithuania | 65 | 250 | 315 | 29 | 29 | 7 | 131 | 100 | 19 | 0.28 | 0.99 |
| 2 | Gineviciene 2 | 199 | 167 | 366 | 95 | 85 | 19 | 83 | 74 | 10 | 0.28 | 0.22 |
| 3 | Jin | 111 | 145 | 256 | 37 | 58 | 16 | 39 | 77 | 29 | 0.47 | 0.42 |
| 4 | Maciejewska Poland | 176 | 684 | 860 | 104 | 65 | 7 | 280 | 314 | 90 | 0.36 | 0.89 |
| 5 | Maciejewska Russia | 538 | 1132 | 1670 | 268 | 226 | 44 | 489 | 505 | 138 | 0.35 | 0.67 |
| 6 | Peplonska | 413 | 457 | 870 | 199 | 178 | 36 | 199 | 213 | 39 | 0.32 | 0.09 |
|  |  |  |  |  |  |  |  |  |  |  |  |  |

* Laplace-corrected values; Gly: glycine; Ser: serine; maf: minor allele frequency in controls; ; K: number designation of the study
