# Supplementary material for: Association of PPARGC1A Gly428Ser (rs8192678) polymorphism with potential for athletic ability and sports performance: A meta-analysis
Source: PLoS One. 2019 Jan 9;14(1):e0200967. doi: 10.1371/journal.pone.0200967 (PMC6326506; doi:10.1371/journal.pone.0200967)
Supplement: S3 Table — (DOCX) [file pone.0200967.s004.docx]

**Meta-analysis on genetic association studies checklist | PLOS ONE**

|  | Item | Section name and paragraph number within manuscript |
| --- | --- | --- |
|  | **Introduction** |  |
| 1 | Provide a detailed justification for the polymorphism studied; if a single polymorphism was analyzed, give details as to why others were not included in the meta-analysis. | Page 4  Lines: 117-124; 146-148; 150-152 |
| 2 | Provide a detailed justification for the population(s) and clinical condition studied. | Page 4  Lines: 125-132 |
|  | **Methods** |  |
| 3 | Provide full details of the search strategy employed; outline the full electronic search strategy–specific combination of keywords and any limits applied- for at least one database. Specify whether synonyms of polymorphisms/genes (e.g. SNP number) were searched. | Page 6  Lines: 165-169 |
| 4 | Report full details on the inclusion and exclusion criteria applied for selecting studies.  Please list the excluded articles and the reasons for exclusion of each article in a supplementary file. | Pages 6-7  Lines: 170-179  Supplementary material S2 lists of excluded articles |
| 5 | Provide details on how the quality of the studies included in the analyses was assessed. | Page 8  Pages: 206-211 |
| 6 | Describe steps taken to contact study authors to identify additional studies and to request missing data. | Page 7  Lines: 191-192 |
| 7 | Describe how environmental effects were adjusted for, if this adjustment was not conducted, outline the reasons for this. | Page 7  Lines: 188-190 |
| 8 | Describe the methods of handling heterogeneity/between-study variance. | Pages 8-9  Lines: 229-238 |
| 9 | Describe how the Hardy-Weinberg equilibrium and linkage disequilibrium were assessed. | Page 7  Lines: 190-191 |
| 10 | Describe and justify the choice of model for the analyses (per-allele vs per-genotype vs genetic model-free, random effects vs fixed effects). | Page 9  Lines: 231-232;  Lines: 236-238 |
| 11 | Describe whether a sensitivity analysis has been completed. | Page 9  Lines: 239-240 |
| 12 | Describe whether an assessment of the effects of population stratification has been conducted. | Page 8  Lines: 229-230 |
| 13 | Describe whether study-specific results have been assessed and if so the reasons for this (e.g. forest plot). | Page 8  Lines: 220-228 |
|  | **Results** |  |
| 14 | Include flow diagram for the studies included in the meta-analysis as the first figure for the manuscript | Page 9  Lines: 246-251 |
| 15 | Report details on allele/genotype prevalence. | Page 10  Lines: 263-266 |
| 16 | Report the effect size estimates and p values for each analysis. | Pages 11-15  Lines: 275-366 |
|  | **Discussion** |  |
| 17 | Discuss the limitations of the meta-analysis, including genotyping errors/bias and publication bias. | Page 21  Lines: 487-498 |
| 18 | If the meta-analysis identifies an association within a subgroup of the population studied but not another, discuss the implications of these results, and if applicable the possibility of subgroup-specific publication bias. | Pages 17-19  Lines: 397-446 |
| 19 | Discuss the suitability of the sample size employed to the research question and the power of the study. | Page 17  Lines: 413-427  Page 21  Lines: 501-502  Page 22  Lines: 521-522 |
