# Supplementary material for: Association of PPARGC1A Gly428Ser (rs8192678) polymorphism with potential for athletic ability and sports performance: A meta-analysis
Source: PLoS One. 2019 Jan 9;14(1):e0200967. doi: 10.1371/journal.pone.0200967 (PMC6326506; doi:10.1371/journal.pone.0200967)
Supplement: S4 Table — (DOCX) [file pone.0200967.s005.docx]

**S4 Table Outlier and modified effects for Ser allele associations with sports performance**

|  |  |  |  |  |  |  |  |  |  |  |  |  |  |  |  |  |  |  |  |
| --- | --- | --- | --- | --- | --- | --- | --- | --- | --- | --- | --- | --- | --- | --- | --- | --- | --- | --- | --- |
|  |  | Test of association | | |  | Test of heterogeneity | | |  |  | Test of association | | |  | Test of heterogeneity | | | Effects of outlier treatment Fs | |
|  | n | OR | 95% CI | P^a^ | SP | P^b^ | I^2^ (%) | AM |  | n | OR | 95% CI | P^a^ | SP | P^b^ | I^2^ (%) | AM | Signi-ficance | Hetero-geneity |
|  |  | PRO | | | | | | |  |  | PSO | | | | | | |  |  |
|  |  |  |  |  |  |  |  |  |  |  |  |  |  |  |  |  |  |  |  |
| All | 16 | 0.84 | 0.67-1.07 | 0.16 | Ds | 10^-5^ | 69 | R |  | 12 | 1.08 | 0.92-1.28 | 0.34 | Fs | 0.41 | 3 | F | RNS | RH |
| Power | 7 | 0.83 | 0.65-1.05 | 0.12 | Ds | 0.31 | 16 | F |  | --- | ------ | ------ | ------ | --- | --- | --- | --- | ---- | ---- |
| Endurance | 11 | 0.82 | 0.95-1.13 | 0.23 | Ds | 0.0003 | 69 | R |  | 9 | 0.95 | 0.79-1.14 | 0.58 | Ds | 0.10 | 40 | F | ---- | ---- |
| Mixed | 6 | 0.79 | 0.52-1.22 | 0.30 | Ds | 0.01 | 66 | R |  | 5 | 0.83 | 0.66-1.05 | 0.12 | Ds | 0.11 | 46 | F | ---- | ---- |
| ***Race*** |  |  |  |  |  |  |  |  |  |  |  |  |  |  |  |  |  |  |  |
| Caucasian | 13 | 0.81 | 0.61-1.08 | 0.15 | Ds | 10^-4^ | 71 | R |  | 9 | 1.13 | 0.92-1.40 | 0.25 | Fs | 0.41 | 3 | F | RNS | RH |
| Asian | 3 | 1.01 | 0.79-1.31 | 0.91 | Null | 0.27 | 24 | F |  | --- | ------ | ------ | ------ | --- | --- | --- | --- | ---- | ---- |
| ***Modified*** |  |  |  |  |  |  |  |  |  |  |  |  |  |  |  |  |  |  |  |
| All | 10 | 0.83 | 0.64-1.08 | 0.17 | Ds | 0.0004 | 70 | R |  | 8 | 0.92 | 0.79-1.07 | 0.27 | Ds | 0.10 | 41 | F | ---- | ---- |
| All > 80% | 5 | 0.68 | 0.50-0.93 | 0.02 | Ds | 0.01 | 70 | R |  | 3 | 0.57 | 0.47-0.68 | 10^-5^ | Ds | 0.17 | 43 | F | ---- | ---- |
| Power | 6 | 0.86 | 0.67-1.10 | 0.22 | Ds | 0.27 | 22 | F |  | --- | ------ | ------ | ------ | --- | --- | --- | --- | ---- | ---- |
| Endurance | 7 | 0.80 | 0.58-1.11 | 0.18 | Ds | 0.009 | 65 | R |  | 6 | 0.90 | 0.73-1.11 | 0.33 | Ds | 0.19 | 33 | F | ---- | ---- |
| Mixed | 4 | 0.71 | 0.41-1.23 | 0.22 | Ds | 0.01 | 72 | R |  | 3 | 0.79 | 0.60-1.03 | 0.09 | Ds | 0.13 | 51 | F | ---- | ---- |
| ***Race*** |  |  |  |  |  |  |  |  |  |  |  |  |  |  |  |  |  |  |  |
| Caucasian | 8 | 0.76 | 0.57-1.03 | 0.08 | Ds | 0.003 | 67 | R |  | 6 | 0.85 | 0.70-1.02 | 0.08 | Ds | 0.11 | 44 | F | ---- | ---- |
| Asian | 2 | 1.09 | 0.83-1.44 | 0.53 | Fs | 0.35 | 0 | F |  | --- | ------ | ------ | ------ | --- | --- | --- | --- | ---- | ---- |
|  |  |  |  |  |  |  |  |  |  |  |  |  |  |  |  |  |  |  |  |

n: number of studies; Modified: ≥ 248 sample size in either case or control; All > 80%: studies with ≥ 248 participants in case and in control; PRO: pre-outlier; PSO: post-outlier; OR: odds ratio; CI: confidence interval; P^a^: P-value for association; P^b^: P-value for heterogeneity; AM: analysis model; R: random-effects; F: fixed-effects; SP: sports performance; Fs: favor SP; Ds: disfavor SP; ORs = 0.99-1.01 were considered null; RNS: retained non-significance; RH: reduced heterogeneity.
