# Supplementary material for: Association of PPARGC1A Gly428Ser (rs8192678) polymorphism with potential for athletic ability and sports performance: A meta-analysis
Source: PLoS One. 2019 Jan 9;14(1):e0200967. doi: 10.1371/journal.pone.0200967 (PMC6326506; doi:10.1371/journal.pone.0200967)
Supplement: S5 Table — (DOCX) [file pone.0200967.s006.docx]

**S5 Table Modified effects for Ser-Gly genotype associations with sports performance**

|  |  | Test of association | | |  | Test of heterogeneity | |
| --- | --- | --- | --- | --- | --- | --- | --- |
|  | n | OR | 95% CI | P^a^ | SP | P^b^ | I^2^ (%) |
|  |  |  |  |  |  |  |  |
| All | 16 | 0.86 | 0.80-0.92 | 10^-4^ | Ds | 0.21 | 21 |
| Power | 7 | 0.86 | 0.75-1.00 | 0.04 | Ds | 0.17 | 34 |
| Endurance | 11 | 0.86 | 0.78-0.95 | 0.003 | Ds | 0.22 | 24 |
| Mixed | 6 | 0.82 | 0.72-0.93 | 0.003 | Ds | 0.52 | 0 |
| ***Race*** |  |  |  |  |  |  |  |
| Caucasian | 13 | 0.84 | 0.77-0.91 | 10^-4^ | Ds | 0.17 | 27 |
| Asian | 3 | 1.00 | 0.81-1.23 | 1.00 | Null | 0.98 | 0 |
| ***Modified*** |  |  |  |  |  |  |  |
| All | 10 | 0.85 | 0.79-0.92 | 10^-4^ | Ds | 0.13 | 35 |
| All > 80% | 5 | 0.83 | 0.76-0.91 | 10^-4^ | Ds | 0.21 | 31 |
| Power | 6 | 0.86 | 0.74-1.00 | 0.06 | Ds | 0.10 | 45 |
| Endurance | 7 | 0.87 | 0.78-0.96 | 0.008 | Ds | 0.24 | 25 |
| Mixed | 4 | 0.80 | 0.69-0.92 | 0.002 | Ds | 0.36 | 7 |
| ***Race*** |  |  |  |  |  |  |  |
| Caucasian | 8 | 0.83 | 0.76-0.91 | 10^-4^ | Ds | 0.12 | 39 |
| Asian | 2 | 1.01 | 0.80-1.26 | 0.95 | Null | 0.90 | 0 |
|  |  |  |  |  |  |  |  |

n: number of studies; Modified: ≥ 248 sample size in either case or control; All > 80%: studies with ≥ 248

participants in case and in control; OR: odds ratio; CI: confidence interval; P^a^: P-value for association;

P^b^: P-value for heterogeneity; SP: sports performance; Ds: disfavor SP; ORs = 0.99-1.01 were considered null.

All comparisons were performed under the fixed-effect model.
